# A Physiologically Based, Multi-Scale Model of Skeletal Muscle Structure and Function

**DOI:** 10.3389/fphys.2012.00358

**Published:** 2012-09-13

**Authors:** O. Röhrle, J. B. Davidson, A. J. Pullan

**Affiliations:** ^1^Institute of Applied Mechanics (Civil Engineering), University of StuttgartStuttgart, Germany; ^2^Cluster of Excellence for Simulation Technology, University of StuttgartStuttgart, Germany; ^3^Engineering Computational Biology Group, School of Computer Science and Software Engineering, The University of Western AustraliaCrawley, WA, Australia; ^4^Auckland Bioengineering Institute, The University of AucklandAuckland, New Zealand

**Keywords:** skeletal muscle mechanics, multi-scale, continuum mechanics, excitation-contraction coupling, motor-unit recruitment, tibialis anterior

## Abstract

Models of skeletal muscle can be classified as phenomenological or biophysical. Phenomenological models predict the muscle’s response to a specified input based on experimental measurements. Prominent phenomenological models are the Hill-type muscle models, which have been incorporated into rigid-body modeling frameworks, and three-dimensional continuum-mechanical models. Biophysically based models attempt to predict the muscle’s response as emerging from the underlying physiology of the system. In this contribution, the conventional biophysically based modeling methodology is extended to include several structural and functional characteristics of skeletal muscle. The result is a physiologically based, multi-scale skeletal muscle finite element model that is capable of representing detailed, geometrical descriptions of skeletal muscle fibers and their grouping. Together with a well-established model of motor-unit recruitment, the electro-physiological behavior of single muscle fibers within motor units is computed and linked to a continuum-mechanical constitutive law. The bridging between the cellular level and the organ level has been achieved via a multi-scale constitutive law and homogenization. The effect of homogenization has been investigated by varying the number of embedded skeletal muscle fibers and/or motor units and computing the resulting exerted muscle forces while applying the same excitatory input. All simulations were conducted using an anatomically realistic finite element model of the tibialis anterior muscle. Given the fact that the underlying electro-physiological cellular muscle model is capable of modeling metabolic fatigue effects such as potassium accumulation in the T-tubular space and inorganic phosphate build-up, the proposed framework provides a novel simulation-based way to investigate muscle behavior ranging from motor-unit recruitment to force generation and fatigue.

## 1 Introduction

Research on investigating and analyzing functional or structural properties of skeletal muscles, e.g., fatigue, injury, aging, or muscle fiber composition, focuses almost entirely on *in vitro* or *in vivo* experiments. The restricted knowledge on the underlying complex mechanisms and their causal correlations often fosters a research environment focusing on mechanisms and components in isolation. Each knowledge gain is invaluable and provides a valuable step toward understanding skeletal muscle mechanics and the musculoskeletal system as a whole. However, if experimentalists worked hand-in-hand with theorists to exploit the dormant power of detailed biophysical computer models, it might be possible to approach this goal much quicker and more efficiently.

Using computer simulations in conjunction with experimental findings can provide an invaluable tool to test and evaluate complex hypothesis and conclusions. Comprehensive *in silico* analysis are able to identify important aspects or correlations needing further insights, and hence provide, *a priori*, valuable information for experimental research.

The limiting factor of combining *in vivo* or *in vitro* experiments with *in silico* ones is often the acceptance and/or the simplicity of existing (detailed) biophysical models. Moreover, in case of analyzing muscle fatigue, injury, or aging of skeletal muscles, the models need to extend beyond modeling “only” skeletal muscle tissue. They need to touch and embrace related research areas and fields. For example, comprehensive skeletal muscle models should also take into account neurophysiological aspects, such as motor-unit recruitment principles, functional aspects of motor unit, and muscle fiber type distributions, sub-cellular mechanism, as well as the mechanical behavior of adjacent tissue, and/or the dynamics of (parts of) the musculoskeletal system.

Current computational models of skeletal muscle models do typically either focus on sub-cellular processes of a (half) sarcomere or on simplified phenomenological relationships mimicking the overall (mechanical) behavior of a single skeletal muscle. Based on the focus of the respective skeletal muscle models, one can divide most of the existing skeletal muscle models into two very broad categories: (i) biophysically based models and (ii) phenomenologically based models. Biophysically based skeletal muscle models calculate the output of skeletal muscle using an analysis of intrinsic physiological properties (e.g., Hodgkin and Huxley, 1952; Huxley, 1957). Phenomenological ones use mathematical representations to describe the relationships between input and output properties (e.g., Hill, 1938; Winters and Stark, 1987) – mainly to describe the mechanical properties of skeletal muscle.

Phenomenological models often use the findings of experimentalists to describe their input-output relationship and hence are less suitable for testing hypothesis and assumptions on the same scale, e.g., the scale of a single skeletal muscle. However, they can provide great insights into the applicability and validity of the input-output relationships by using these models on larger scales, e.g., the musculoskeletal system. Hill-type skeletal muscle models (e.g., Hill, 1938; Winters and Stark, 1987) appeal, for example, to a simple mechanical system, e.g., often to a three-element spring-dashpot system, to describe the force-generating properties of skeletal muscles. Clearly, they cannot be used to investigate intrinsic properties of skeletal muscle force generation, but they can be used to test the force-generating relationships of single skeletal muscles in the overall musculoskeletal system, e.g., to answer the question of whether experimentally derived force relationships are sufficient to act against physiological loads. Hill-models, like any other model, do exhibit modeling deficiencies; here for example on the organ system scale (the musculoskeletal system), where they cannot account for the three-dimensional structural complexity of an individual muscles and therefore cannot account for its interaction with surrounding tissue. Further, the model reduces a skeletal muscle to a force acting between an insertion point and an origin point, i.e., to an one-dimensional object.

Recently, full three-dimensional models of skeletal muscles have been created by a number of authors (Johansson et al., 2000; Oomens et al., 2003; Blemker et al., 2005; Lemos et al., 2005; Röhrle and Pullan, 2007; Böl and Reese, 2008). These models have led to a fuller understanding of muscle force distributions. The three-dimensional nature of the models resulted in the ability to analyze dynamic changes to the line of muscle action that cannot be determined from the more common one-dimensional models (Röhrle and Pullan, 2007), as well as elucidating possible causes of on-linear muscle strains (Blemker et al., 2005). These models are all based on the principles of continuum mechanics and result in macroscopic models that do not explicitly include any information from finer scales, e.g., the cellular level. This finer detail, however, is required to represent the changes in muscle properties as a result of disease or injury. The continuum representation also prohibits the use of functional information, which is important for rehabilitation techniques (e.g., functional electrical stimulation). Examples of such functional information include, motor-unit distributions, fiber firing rates, and different locations of fiber types. Furthermore, continuum-mechanical models cannot account for physiological changes, e.g., fatigue induced changes to mechanical output. Within these continuum-mechanical models, fatigue can essentially only be considered in a phenomenological way (e.g., Böl et al., 2009). This however, is of limited use, if one wants to jointly investigate functional and physiological aspects in conjunction with experimental studies.

More recently, researchers have focused on extending the continuum-mechanical models by taking into account the underlying electrophysiology. The aim is to drive the mechanics of full three-dimensional skeletal muscle models by electro-physiological principles, i.e., Fernandez et al. (2005) and Böl et al. (2011). These models, while providing more realistic responses, however do not fully represent the detailed electrophysiology (electrically isolated, independent motor units) of skeletal muscle.

The shortcoming of linking an electrical stimuli at the neuromuscular junction with mechanical output through a biophysically based cellular model, which is also capable of mimicking muscle fatigue, was first addressed in Röhrle et al. (2008) and then further extended to a specific muscle geometry in Röhrle (2010). Röhrle et al. (2008) also demonstrated on a cube that the proposed homogenization methodology, which links the output of a detailed biophysical model to a continuum-mechanical constitutive law, is feasible and delivers for different finite element (FE) discretizations FE convergence rates, which are comparable to a continuum-mechanical model. The key difference of Röhrle et al. (2008) and Röhrle (2010) to all other existing electromechanical skeletal muscle models (e.g., Fernandez et al., 2005; Böl et al., 2011) is the fact that in the proposed model the active contribution within the continuum-mechanical constitutive law is directly coupled to a detailed skeletal muscle model of the (sub-)cellular processes (Shorten et al., 2007) through a multi-scale constitutive law.

This paper aims to extend the framework proposed by Röhrle et al. (2008) and Röhrle (2010) to include a much larger array of anatomical and physiological properties; properties that are the key to modeling the underlying mechanisms behind many diseases and rehabilitation techniques. The main focus therefore is to provide the fundamental algorithms and modeling considerations for incorporating muscle fiber and motor-unit distributions within skeletal muscles and the ability to link a neurophysiological model of motor-unit recruitment to the electromechanical model. The methodology, here enhanced by structural and functional components, is applicable to any skeletal muscle. Herein, the feasibility of developing such a framework is demonstrated on the tibialis anterior (TA) muscle. The result is one of the most advanced and detailed skeletal muscle model currently available.

Such a detailed model becomes necessary, if one strives to obtain a deeper understanding of skeletal muscle function during muscle recruitment and to obtain a better understanding of how the interplay between muscle fiber recruitment mechanisms and mechanical force generation can be affected by alterations to the underlying muscle properties. This model will be able to provide for many different fields a framework capable of investigating injuries and disease processes that affect skeletal muscle encompassing the cellular composition, the functional recruitment processes, and the gross mechanical structure. Moreover, the proposed model will have potential to generate impact to the general field of computational neuroscience, in which all state-of-the-art recruitment models link their findings to simplistic muscle force models neglecting any kind of spatial characteristics. In general, the modular structure of the proposed framework shall allow easy model adaptations, such that the overall framework can be successfully applied to many different fields of musculoskeletal research.

## 2 Materials and Methods

The skeletal muscle framework proposed in Röhrle et al. (2008) and Röhrle (2010) focused on developing the underlying methodology to embed electrically isolated skeletal muscle fibers within a three-dimensional skeletal muscle geometry and to link their cellular behavior to mechanical output. Based on the same tibialis anterior mesh as introduced in Röhrle (2010; cf. Section 2.1.1), the modeling framework presented herein is extended by the following anatomical and physiological enhancements:

1. Grouping of skeletal muscle fibers into functional units, i.e., the motor units (cf. Section 2.1.2),
2. fiber type segregation of motor units into fast- or slow-twitch type (Henneman and Olson, 1965; Wuerker et al., 1965; Andreassen and Arendt-Nielsen, 1987; Monti et al., 2001; Duchateau et al., 2006),
3. a physiological distribution of fibers within motor units (Enoka and Fuglevand, 2001),
4. an anatomically based spatial distribution of fibers into motor units (Bodine-Fowler et al., 1990; Roy et al., 1995; Monti et al., 2001),
5. motor-unit territory sizes proportional to the number of fibers per motor unit (Fuglevand et al., 1993; Roy et al., 1995; Yao et al., 2000; Monti et al., 2001),
6. recruitment of each skeletal muscle fiber of a particular motor unit through the neural input of an α-motor neuron (cf. Section 2.4), and
7. controlling the muscle force output by recruitment and rate coding of motor units (Fuglevand et al., 1993).

In summary, the proposed modeling framework encompasses (i) an anatomically based, three-dimensional mechanical model of a skeletal muscle, (ii) the electrophysiology of a single muscle fiber, (iii) the coupling of the electrophysiology (cellular) to the mechanical description through a cellular based multi-scale constitutive law, and (iv) the mechanical response of an entire muscle due to neural stimulation using a phenomenological model of motor-unit recruitment.

### 2.1 A three-dimensional, anatomically based muscle geometry

#### 2.1.1 The FE mesh and the embedding of the muscle fibers

**Figure 1:**
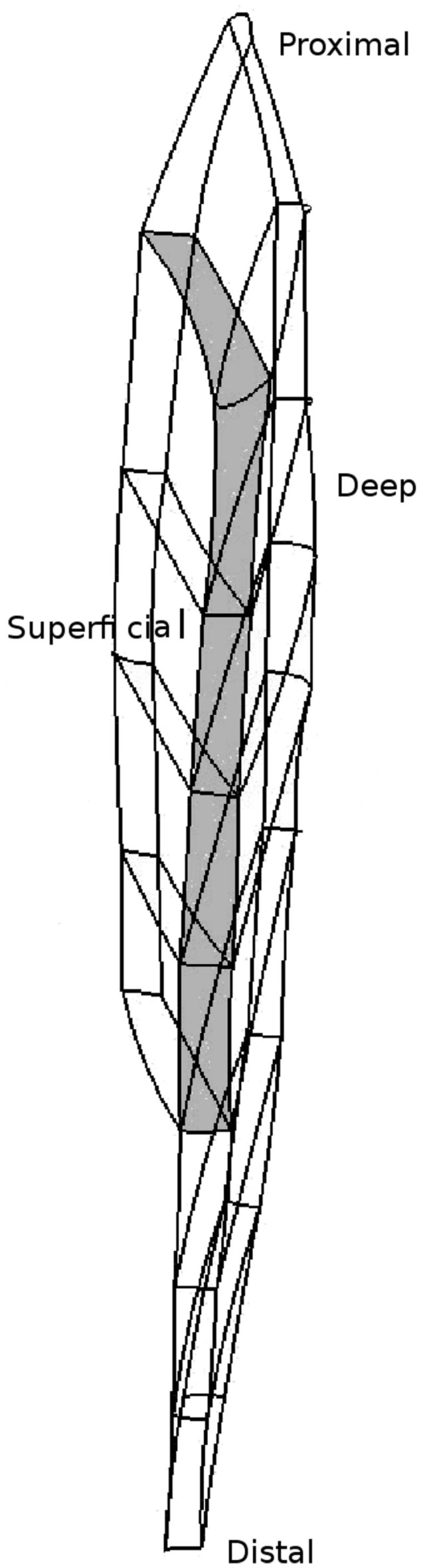
**The tri-quadratic FE mesh of the tibialis anterior (TA)**. The shaded section represents the element boundaries that are aligned with the aponeurosis separating the superficial and deep compartments of the TA.

The basis of this framework is a three-dimensional, anatomically based representation of the TA muscle. To generate the geometrical representation of the muscle, two-dimensional photographic slices of the Visible Human male data set (Spitzer and Whitlock, 1998) were manually digitized to create a three-dimensional data cloud. The data cloud was used to fit a three-dimensional quadratic Lagrange FE mesh using a least-squares minimization algorithm. For details about the fitting algorithm, the reader is referred to Bradley et al. (1997). The generated FE mesh distinguishes between the superficial and deep compartments of the TA, which are separated through an aponeurosis, and is depicted in Figure 1. To simplify the embedding of the muscle fibers, the FE mesh of the TA has been constructed in such a way that the muscle fibers within the TA will be aligned with a direction of the local FE coordinate system of each element. All fibers within the three-dimensional FE mesh are represented as one-dimensional lines consisting of evenly spaced grid points. Moreover, all fibers are assumed to have the same cross-sectional fiber area. The necessary fiber angle data for achieving the respective fiber angles within the FE mesh are taken from the fiber pennation angle data published in Lansdown et al. (2007). The pennation angle measurements in Lansdown et al. (2007) are based on *in vivo* diffusion-tensor magnetic resonance imaging (DT-MRI).

#### 2.1.2 Functional grouping of muscle fibers

Within this work, the distribution of muscle fibers per motor unit is based on the work of Enoka and Fuglevand (2001). They assume that the number of muscle fibers per motor unit exhibits an exponential distribution: many motor units exert small forces, i.e., contain a small number of muscle fibers, and relatively few motor units exert large forces, i.e., contain a large number of muscle fibers. Such an exponential distribution of muscle fibers per motor unit can be determined for a fixed number of total muscle fibers, *f ^M^*, by computing for each motor unit *i* the number of associated fibers through

(1) $$f_{i}=f_{1}\cdot \exp\left(\frac{i-1}{m}\cdot \ln\;f_{\text{ratio}}^{\text{m}∕1}\right),$$

while ensuring that $\sum_{i=1}^{m}\;f_{i}=f^{M}.$ In equation (1), $f_{\text{ratio}}^{\text{m}∕1}$ determines the ratio between the number of muscle fibers of the largest motor unit, *f_m_*, and the smallest motor unit, *f*_1_. As the number of fibers within this work is dictated by the number of embedded fibers, equation (1) has to be solved iteratively. As the solution of equation (1) typically results in non-integer values, each *f_i_* is rounded to its nearest integer. To fulfill the constraint that the sum of all assigned muscle fibers has to equal *f ^M^*, the number of fibers of the last motor unit is adjusted by $f_{m}=f^{M}-\sum_{i=1}^{m-1}\;f_{i}.$

Each motor unit is assumed to be composed of either fast- or slow-twitch muscle fibers, with smaller motor units composed of slow type muscle fibers (Henneman et al., 1965b; Wuerker et al., 1965). The proportion of slow versus fast muscle fibers is specified by *p*^s/f^ (about 70% of a human TA’s muscle fibers are of slow type). Hence, by defining the first *s* motor units to be slow, and the last *m* − *s* motor units to be fast, one can determine for a pre-defined *p*^s/f^ the number of *s* slow motor units by the following relationship:

(2) $$s=\underset{l}{\text{argmin}}\left|\frac{\sum_{i=1}^{l}\;f_{i}}{\sum_{i=l+1}^{m}\;f_{i}}-p^{\text{s/f}}\right|.$$

The assignment of embedded fibers to a specific motor unit is achieved by defining motor-unit territories within the mesh and then assigning random fibers within the territories to a motor unit. As fibers within a motor unit are defined to be of the same physiological type (i.e., fast or slow type) and as muscle fibers of a certain type are preferentially located in a certain region of the muscle (Polgar et al., 1973; Henriksson-Larsén et al., 1983), additional weighting centers $W^{t}$ with *t *∈ {*s, f*} are introduced. The distance between a weighting center, $W^{t}$, and a particular center of a motor-unit territory, $C_{i}$, is assumed to be normal distributed, i.e., $N\left(\mu_{t},\sigma_{t}^{2}\right).$ The mean, μ*_t_*, is given by the location of the weighting center, $W^{t}$, while the variance is determined based on the location of the muscle fiber center points, $f_{k}^{c}$, and is therefore dependent on the anatomical arrangement of the muscle fibers and on the muscle geometry itself. The two variances for *t *∈ {*s, f*} are given by

(3) $$\sigma_{t}=\frac{2\omega_{t}}{3}\left(\underset{k}{\max}\left\{d_{k,t}\right\}-\underset{k}{\min}\left\{d_{k,t}\right\}\right),$$

where *k* = 1,…, *f ^M^* is an index for a particular fiber, ω*_t_* denotes a scaling factor, and

(4) $$d_{k,t}=∥f_{k}^{c}-W^{t}{∥}_{2}$$

defines the Euclidean distance between the muscle fiber midpoint of the *k*th-fiber, $f_{k}^{c},$ and the respective weighting center $W^{t}$. The scaling parameter, ω*_t_*, is introduced to account for cases in which the motor-unit territory centers are defined to be in close proximity to the weighting center or for cases in which this condition shall be relaxed. In case that no weighting points can be specified, or if one prefers to distribute the motor-unit territory center points over the entire geometry of the muscle, the normal random distribution is substituted by an uniform random distribution. Within this work, the scaling parameter, ω*_t_*, is chosen to be 1.

The motor-unit territory centers, $C_{i}$ for *i* = 1,…, *f ^M^*, are selected by choosing *s* random numbers $X_{i}^{s}∼N\left(\mu_{s},\sigma_{s}\right)$ and *m* − *s* random numbers $X_{i}^{f}∼N\left(\mu_{f},\sigma_{f}\right),$ and by determining the closest muscle fiber midpoint

(5) $$C_{i}=\underset{f_{k}^{c}\in F}{\text{argmin}}{\left\|f_{k}^{c}-\left(\underset{k}{\min}\left\{d_{k,t}\right\}+X_{i}^{t}\right)\right\|}_{2}$$

with *i* = 1,…, *m* and *t *∈ {*s*, *f*} accordingly.

To determine the spatial distribution of muscle fibers for each motor unit *i*, single fibers from the pool of all available fibers, which is denoted by $M^{r}$ and coincides with F prior of selecting the first fiber, are successively selected and removed from the set of all remaining fibers, $M^{r}$. The selection algorithm for $F_{i}$, which is the set of all muscle fibers numbers associated with motor unit *i*, is described in the following in more detail: First, all potential muscle fiber midpoints for motor unit *i* are determined by:

(6) $$P_{i}=\left\{k|f_{k}\in M^{r}\text{ and }{\left\|f_{k}-C_{i}\right\|}_{2}\leq R_{i}\right\},$$

where the radius,

(7) $$R_{i}=\sqrt{\frac{f_{i}}{\rho_{i}\cdot \pi }},$$

denotes a spatial constraint depending on the number of fibers, *f_i_*, and the fiber density, ρ*_i_*, of the respective motor-unit territory. The fiber density of a motor-unit territory, ρ*_i_*, is based on physiological data (Roy et al., 1995; Monti et al., 1999). A total of *f_i_* muscle fiber midpoints are randomly selected and removed from the sets $P_{i}$ and $M^{r}$, i.e., $P_{i}$ : = $P_{i}$ − {*k*}, $M^{r}$ : = $M^{r}\;-\;${*k*}, and $F_{i}$ : = $F_{i}$ ∪ {*k*}. Note, in some cases, the choice of *R_i_* might be too restrictive resulting in an initial set $P_{i}$ (before selecting any muscle fibers for motor unit *i*) that does not contain enough elements, i.e., card ($P_{i}$) = |$P_{i}$| ≤ *f_i_*. In this case, the radius, *R_i_* is successively increased by 5% until the set of potential assignable muscle fibers, $P_{i}$, is sufficiently large. The same procedure is repeated *m*-times, starting from the smallest motor unit and ending with the largest one. Following this procedure, all muscle fibers are uniquely assigned to a particular motor unit.

### 2.2 Modeling the physiology of a skeletal muscle fiber

Although fibers within a skeletal muscle are mechanically coupled, from an electro-physiological point of view, they are independent. We assume that the activity of all fibers in a motor unit can be modeled as identical and that all fibers are innervated at their midpoint. Hence, it is sufficient to model the activation of a single muscle fiber per motor unit, and use its output for all associated ones. The overall electro-physiological model of a single muscle fiber can be split up in two parts: (i) the first part focuses on modeling the (sub-)cellular behavior at a particular point along the muscle fiber and (ii) the second part focuses on the propagation of the AP along its muscle fiber. The first part can be described by a Hodgkin–Huxley-like cellular model and is elucidated in more detail in Subsection 2.2.1. The propagation of the AP along a single skeletal muscle fiber is an interplay between the cellular response of the muscle fiber at a particular location and of the propagation of the electrical signal (AP). The homogenization of the intra- and extracellular processes and the propagation of the AP along the muscle fiber can be modeled using the bidomain equations, which are described in detail in Subsection 2.2.2.

#### 2.2.1 The cellular model

Many electro-physiological models describing various aspects of (sub-)cellular processes have been introduced. The specific skeletal muscle cell model used within this work is, like in Röhrle et al. (2008) and Röhrle (2010), the model developed by Shorten et al. (2007). This cell model describes by means of two separate sets of parameters the electro-physiological behavior of a (half-)sarcomere within slow- and fast-twitch type skeletal muscle fibers using a Hodgkin–Huxley-like description. The resulting system of coupled ordinary differential equations (ODEs) has been implemented using CellML, which is a Markup-Language specifically designed to describe cellular processes (cf. Lloyd et al., 2008). The model can be downloaded^1^ from the CellML-website, www.cellml.org.

In brief, the Shorten et al. (2007) model can be described as an amalgamation of cell models, which each individually describe parts of the muscle cell physiology. The model contains descriptions of cellular processes such as the sarcolemmal membrane potential, excitation-contraction coupling, and the dynamics of the actin-myosin crossbridges. In modeling the membrane potentials, the sarcolemmal and T-tubular membranes are represented separately allowing the representation of fatigue effects through potassium accumulation in the T-tubular space. In addition, the effects of inorganic phosphate build-up are modeled to represent metabolic fatigue properties. The actin-myosin crossbridge model is an “eight-state model,” in which the eight “states” can be associated with regulatory units on the thin filaments and, therefore, can be are related to different states of the troponin-tropomyosin complex. Specifically, the different states within this model are distinguished into six detached states and two attached states, i.e., the concentration of attached myosin crossbridges during the pre-powerstroke state, *A*_1_, and the concentration of attached myosin crossbridges during the post-powerstroke state, *A*_2_. The parameters *A*_1_ and *A*_2_ will be used later within the multi-scale constitutive law describing the overall electromechanical behavior of the skeletal muscle.

The Shorten et al. (2007) cell model is selected, because it represents the cellular properties of skeletal muscle fibers from action potential (AP) activation right through to the crossbridge dynamics. The main advantage of this model is that the entire chain of processes allows for a more physiologically realistic representation of complex cellular behavior such as membrane fatigue, metabolic fatigue, force summation, potentiation, and the catch-like effect (Shorten et al., 2007). Further, it allows the modeling of different muscle fiber types, e.g., fast and slow-twitch muscle, without the necessity of separate cell models.

#### 2.2.2 The bidomain equations

The behavior of AP propagation in biological tissue is typically modeled using the bidomain equations (Pullan et al., 2005), which are a set of coupled reaction-diffusion equations. The reactive part stems from the cellular behavior, while the diffusive part describes the propagation of the AP. For examples of implementation of ODE models of cellular activity refer to Fernandez et al. (2005), Kim et al. (2007), and Röhrle and Pullan (2007).

Within this work, we assume a fiber diameter of 80 μm, which is within the range of a human fiber diameter of 80–100 μm (e.g., Lexell et al., 1986; Sjöström et al., 1991; Miller et al., 1993) motivating the surface-to-volume ratio of 50 mm^−1^. The capacitance of the membrane was set at 0.01 μFmm^−2^ for the fast-twitch fibers and 0.0058 μFmm^−2^ for the slow-twitch fibers (cf. Shorten et al., 2007). The intracellular and extracellular conductivity tensors are, in the case of solving the bidomain equations for a one-dimensional fiber, scalars and are chosen to be σ*_i_* = 0.893 mSmm^−1^ (Bryant, 1969) and σ*_e_* = 0.67 mSmm^−1^ (Schwann and Kay, 1957; Rush et al., 1963), respectively.

The bidomain equations are discretized in space using linear Lagrange finite elements and the resulting system of ODEs is then solved using LSODA (Hindmarsh, 1982; Petzold, 1983). The key electro-physiological parameters that will be used later within a multi-scale constitutive law (cf. Section 2.3.1) are the concentration of actin-myosin crossbridges in the pre- and post-powerstroke, *A*_1_ and *A*_2_.

### 2.3 The continuum-mechanical skeletal muscle model

This work appeals to the same mechanical model as proposed and used in Röhrle et al. (2008) and Röhrle (2010). For completeness, however, a brief overview is given in the following.

The continuum-mechanical model proceeds form the local form of the balance of linear momentum,

(8) $$\rho \;\ddot{\text{x}}=\text{div}T+\rho \;b\;,$$

where ρ denotes the mass density, **x** is a material point position in the current configuration and $\ddot{\text{x}}$ its second time derivative, **b** are the body forces, and **T** denotes the Cauchy stress tensor. By assuming quasi-static conditions and small body forces (in comparison to the forces generated by the muscle), the local form of the balance of linear momentum reduces to div **T** = 0. For biological tissues, it is often advantageous to express the stress-strain relationship with respect to anatomical features, which are typically defined in the reference state. To do so, one can express the Cauchy stress tensor in terms of the second Piola–Kirchhoff stress tensor via the push-forward operation **T** = *J*^−1^**FSF**^−1^, where *J* = det **F** and **F** = ∂x/∂X is the deformation gradient tensor mapping points between the reference configuration, **X**, and the current configuration, **x** (deformed state). The strain is typically measured by the Green strain tensor

(9) $$E=\frac{1}{2}\left(F^{T}F-I\right)=\frac{1}{2}\left(C-I\right),$$

where **C** is the right Cauchy–Green deformation tensor and **I** is the identity tensor. The relationship between stress and strain can be specified for hyperelastic materials by a strain energy function *ψ*. A detailed introduction and overview to non-linear continuum mechanics and tensor analysis can be found in Holzapfel (2004).

A skeletal muscle tissue’s overall mechanical behavior can be modeled by distinguishing between an passive and an active behavior. During contraction (the active part) the muscle generates a contractile force in the longitudinal direction, which is (locally) described by a vector *a*_0_. The material behavior of the passive part is described through the mechanical behavior of the tissue’s ground matrix, i.e., the extracellular matrix consisting of a network of collagen, fat, etc. Given the mechanical behavior’s additive nature, the free energy of the entire muscle tissue, ψ^muscle^, can be written as the sum of the free energy of the ground substance, ψ^matrix^, and the free energy of the active part, *ψ*^active^:

$$\begin{array}{llll} \psi^{\text{muscle}}\left(C,a_{0}\right)= & \psi^{\text{matrix}}\left(C,f^{\text{passive}}\left(\lambda \right),a_{0}\right) & & \\ & +\psi^{\text{active}}\left(C,a_{0},\alpha ,f^{\text{active}}\left(\lambda \right)\right), & & \text{(10)} \end{array}$$

where α ∈ [0, 1] is an internal variable that describes the level of activation, $\lambda =\sqrt{a_{0}\cdot Ca_{0}}$ is the fiber stretch, and *f* ^passive^(λ)and *f* ^active^(λ) are the normalized force-length relationships. For the active part, *f* ^active^(λ) describes the overlap of actin and myosin and hence the ability to generate tension through crossbridge dynamics. The normalized force-length relationship is a commonly used tool to incorporate the physiological behavior of fiber stretch in purely mechanical models (e.g., Blemker et al., 2005; Röhrle and Pullan, 2007; Böl and Reese, 2008).

Differentiating the free energy with respect to **C** and assuming a simple and isotropic Mooney–Rivlin-type material behavior for the ground matrix, results in the following definition of the second Piola–Kirchhoff stress tensor

$$\begin{array}{llll} S^{\text{muscle}}= & \underset{=:\;S^{\text{iso}}}{\underset{︸}{c_{1}I+c_{2}\left(I_{1}I-C\right)-p\sqrt{I_{3}}C^{-1}}} & & \\ & +\underset{=:\;S^{\text{aniso}}}{\underset{︸}{\left[\frac{\sigma_{\text{pass}}^{ff}}{I_{4}}f^{\text{passive}}\left(I_{4}\right)\right]\left(a_{0}⊗a_{0}\right)}} & & \\ & +\underset{=:\;S^{\text{active}}}{\underset{︸}{\alpha \left[\frac{\sigma_{\text{ten}}^{ff}}{I_{4}}f^{\text{active}}\left(I_{4}\right)\right]\left(a_{0}⊗a_{0}\right)}}, & & \text{(11)} \end{array}$$

where

$$\begin{array}{llll} & I_{1}=tr\left(C\right), & & \text{(12)}\\ & I_{2}=\frac{1}{2}\left[{\left(tr\left(C\right)\right)}^{2}-tr\left(C^{2}\right)\right], & & \text{(13)}\\ & I_{3}=detC, & & \text{(14)} \end{array}$$

are the standard invariants and invariant

(15) $$I_{4}=a_{0}\cdot Ca_{0}$$

is associated with the fiber stretch in the current configuration, $\sigma_{\text{pass}}^{ff}=\sigma_{\text{ten}}^{ff}=0.03\;\text{MPa}$ are the maximal passive and active stiffness in the along-the-fiber direction, *p* is the hydrostatic pressure and **S**^matrix^ = **S**^iso^ + **S**^aniso^. The dyadic product, **P**, between two three-dimensional vectors is defined by

(16) $$P=u⊗v=\left(\overset{3}{\underset{i=1}{\sum }}\;u_{i}e_{i}\right)⊗\left(\overset{3}{\underset{j=1}{\sum }}\;v_{j}e_{j}\right)=\overset{3}{\underset{i,j=1}{\sum }}\;u_{i}v_{j}e_{i}⊗e_{j},$$

where

(17) $$e_{i}⊗e_{j}=e_{i}e_{j}^{T}=\;\left[\;\;\begin{array}{c} e_{i,1}\\ e_{i,2}\\ e_{i,3} \end{array}\;\right]\;\left[e_{j,1},e_{j,2},e_{j,3}\right]=\;\left[\;\;\begin{array}{ccc} e_{i,1}e_{j,1} & e_{i,1}e_{j,2} & e_{i,1}e_{j,3}\\ e_{i,2}e_{j,1} & e_{i,2}e_{j,2} & e_{i,2}e_{j,3}\\ e_{i,3}e_{j,1} & e_{i,3}e_{j,2} & e_{i,3}e_{j,3} \end{array}\;\right]\;.$$

At each point, the local fiber direction, **a**_0_, can be expressed in terms of the global basis spanning the overall world coordinate system, i.e., **e***_i_*, *i *= 1,2,3. Hence, the dyadic product of the local fiber orientation describes the contribution of the fiber orientations to the overall second Piola–Kirchhoff stress tensor in terms of the global coordinate system. In the special case that the local fiber direction is aligned with the first basis vector of the global coordinate system, i.e., **a**_0 _= **e**_1 _= [1,0,0], the tensor resulting from the dyadic product results into a tensor **a**_0_ ⊗ **a**_0_ with a 1 in (1,1)-component and zeros for all other components. Hence, the fiber will only have a contribution to the (1,1)-component of the second Piola–Kirchhoff stress tensor.

The mechanical model itself is based on solving the governing equations of finite elasticity theory using the FE method. The unknowns describing the displacements are discretized using tri-quadratic Lagrange FE basis functions whilst the unknowns for the hydrostatic pressure are discretized by linear FE basis functions. Solving for the mechanical deformation due to skeletal muscle activity or due to a change in the muscle attachment location (i.e., movement of the bone) requires the evaluation of the second Piola–Kirchhoff stress tensor.

#### 2.3.1 A multi-scale constitutive law

The continuum-mechanical constitutive law does not yet contain any information from the smaller scales such as the cellular level. For a given time t, the activation level, *α*, in equation (11) is substituted by cellular variables, i.e., *A*_1_ and *A*_2_. Moreover, the mechanical description of the contractile response is split into two parts: the first one is based on the generation of tension in the post-powerstroke state and the second one is related to the change of passive stiffness due to the attached crossbridges in the pre- and post-powerstroke states. As both mechanisms act in the direction along a muscle fiber, the second Piola–Kirchhoff stress tensor for the multi-scale constitutive law can be expressed in terms of cellular parameters by

$$\begin{array}{llll} & S^{\text{muscle}}=S^{\text{iso}}+S^{\text{aniso}} & & \\ & \;+\left[\frac{A_{1}+A_{2}}{c_{\text{trop}}}\;\frac{\sigma_{\text{pass}}^{ff}}{I_{4}}f^{\text{active}\;}\left(\lambda \right)+\frac{A_{2}}{A_{2}^{\text{max}}}\;\frac{\sigma_{\text{ten}}^{ff}}{I_{4}}f^{\text{active}\;}\left(\lambda \right)\right]\left(a_{0}⊗a_{0}\right), & & \end{array}$$

where *c*_trop_ is the normalization factor and is the maximum possible amount of troponin within the cellular model (here: *c*_trop_ = 140) and $A_{2}^{\text{max}}$ presents the maximal concentration of attachable crossbridges in the post-powerstroke. Hence, the term containing $A_{2}∕A_{2}^{\text{max}}$ is comparable to *α* in equation (11), while the term containing (*A*_1_ + *A*_2_)/*c*_trop_ relates to an additional passive tension due to the activation.

#### 2.3.2 Upscaling

From a computational point of view, the mechanical model of the skeletal muscle cannot be discretized with the same fine resolution as the embedded fibers due to accuracy constraints dictated by solving the system of ODEs describing the cellular processes. This mismatch in mesh resolutions requires a homogenization procedure to upscale the fine-grid solutions of the cellular variables to the nearest Gauss points of the mechanical FE mesh, at which the second Piola–Kirchhoff stress tensor is evaluated. This is done by computing in the vicinity of each Gauss point the average concentration of attached crossbridges. This homogenization procedure has been proposed and validated in Röhrle et al. (2008).

### 2.4 Motor-unit recruitment

Motor units are recruited in an order determined by the number of muscle fibers in each motor unit, the so-called size principle (Henneman and Olson, 1965; Henneman et al., 1965a,b). The number of active motor units and their firing frequency is modulated as a result of the excitatory drive coming from the motor cortex. This framework assumes that the drive to each motor unit in the pool is equal (De Luca et al., 1982; Yao et al., 2000), though this is still an unresolved issue (Heckman and Binder, 1988; Heckman, 1994). The activation of each motor unit is calculated using a method derived by Fuglevand et al. (1993). Briefly, the recruitment of a specific motor unit and its resulting firing frequency is dependent on a single pre-defined variable *E*(*t*), where, 0 ≤ *E*(*t*) ≤ 1, is a user defined activation parameter. If *E*(*t*) is greater than the recruitment threshold of motor unit *i*, then motor unit *i* becomes active with a firing rate which is linearly dependent on *E*(*t*). The recruitment thresholds of the motor units are defined to be exponentially varying over the pool of motor units

(18) $$T_{i}^{\text{excite}}=\exp^{i\cdot \left(ln\left(RR\right)∕m\right)},$$

where *T_i_* is the recruitment threshold of motor unit *i*, *RR* is the range of recruitment thresholds in the motor pool, and *m* is the number of motor units. Active motor units, i.e., $E\left(t\right)\;\geq \;T_{i}^{\text{excite}},$ are assumed to increase their firing rate linearly from the minimum firing rate to the maximum firing rate. Although it is possible to model variations in minimum and maximum firing rates over the motor pool (Fuglevand et al., 1993), all simulations within this work assume for each active motor unit an uniform minimum firing rate of 8 Hz and a maximum firing rate of 40 Hz. The minimum firing rate is based on the average reported rates in Grimby and Hannerz (1977), Bellemare et al. (1983), Broman et al. (1985), Kamen and Du (1999), McNulty and Cresswell (2004), and Do and Thomas (2005), while the maximum is based on the average value reported in Bellemare et al. (1983) and Enoka and Fuglevand (2001). Hence, the firing times for motor unit *i* can be computed by the following equation:

(19) $$t_{i}^{\text{next}}=t_{i}^{\text{last}}+\frac{1}{\left(1+\eta \cdot c_{\nu }\right)\left(g_{e}\cdot \left[E\left(t_{i}^{\text{next}}\right)-T_{i}^{\text{excite}}\right]+F_{i}^{\text{min}}\right)},$$

if $E\left(t\right)\;\geq \;T_{i}^{\text{excite}}.$ In equation (19), $t_{i}^{\text{last}}$ is the point in time motor unit *i* has fired last, *η* a Gaussian-distributed random number mimicking the natural variability of motor-unit activation, *c_ν_* a coefficient of variation, *g_e_* the gain of motor unit *i*, and $F_{i}^{\text{min}}$ the minimum firing rate of motor unit *i*. The exact parameter values for equation (19) are given in Table 3.

The firing times of each motor unit are used in conjunction with the one-dimensional fiber models to produce the cellular output for each motor unit. It is important to note that each muscle fiber of a specific motor unit has approximately the same behavior as they are innervated by the same motor neuron and are of the same physiological type. Hence, the distribution of the pre- and post-powerstroke concentrations, i.e., the cellular variables *A*_1_ and *A*_2_, are computed for all fibers and serve, after homogenization, as input to the multi-scale constitutive law. Therefore, a change in the state of activation or a displacement boundary conditions at the end of the muscle causes a change in the muscles mechanical state and, hence, the exerted muscle force of the overall skeletal muscle.

## 3 Results

### 3.1 Muscle fiber distribution within the TA

**Figure 2:**
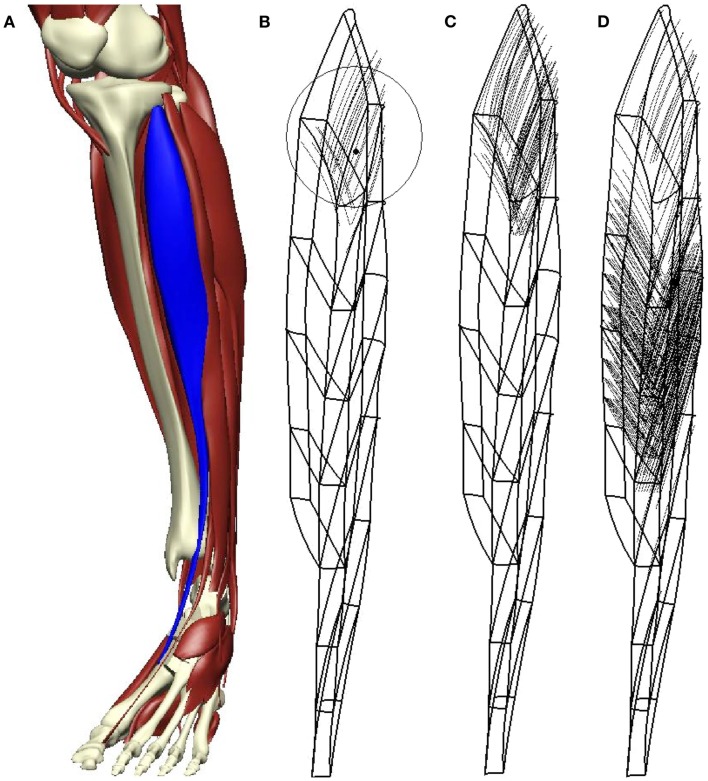
**Subfigure (A) shows the location of the TA within the lower limb; (B–D) show the muscle fibers associated with motor units 1, 5, and 10**. Further **(B)** depicts the motor-unit territory center midpoint, $C_{1}$, for motor unit 1 and (the sphere with) radius *R*_1_ that was used to select the fibers for motor unit 1.

A particular choice of muscle fiber distribution for the TA, which will be used for most numerical investigations within this section, is depicted in Figure 2. The allocation of the muscle fibers to particular motor units has been carried out as described above. A (homogenized) muscle fiber diameter of 2000 μm has been assumed resulting in a total of 903 muscle fibers for the TA muscle geometry depicted in Figure 1. Further, within the muscle-fiber-to-motor-unit-allocation algorithm described above, a total of 30 motor units and a ratio between the number of fibers of the largest and the smallest motor unit of 10, i.e., $f_{\text{ratio}}^{\text{m}∕1}=10$ in equation (1), is considered. The discretization with a grid spacing of 0.0625 mm results in a total of about 50,000 grid points, at which cellular variables need to be computed/assigned. The grid spacing of 0.0625 mm is justified in a convergence study of the AP propagation speed, which is presented within Section 3.2. Figure 2 depicts the anatomical location of the TA (in blue), the muscle fibers associated with motor units 1, 5, and 10, as well as the motor unit territory center $C_{1}$ of the first motor unit. This muscle fiber allocation provides the basis for the remaining simulations, except for the simulations considering a total of 10 and 50 motor units in Section 3.4.

### 3.2 Verification of computational parameters

The contractile properties of the multi-scale constitutive law are directly linked to the electro-physiological behavior of single skeletal muscles. Hence, it is of importance to properly model the physiological behavior of the AP propagation, i.e., the shape of the AP and its conduction velocity along the fiber. All single muscle fiber simulations carried out within this section aim to determine the appropriate temporal and spatial discretization parameters resulting in a correctly simulated AP propagation. The parameters for the bidomain equations are given in Table 1. The values for the Shorten et al. model are outlined in Shorten et al. (2007).

**Table 1:** **Values for the fiber model (bidomain equations)**.

Variable	Value	Description	Sources
*A_m_*	50 mm^−1^	Surface-to-volume ratio	Calculated based on a cell diameter of 80 μm: cf. Lexell et al. (1986)
			Miller et al. (1993)
			Sjöström et al. (1991)
*C_m_*	0.01 mFmm^−2^	Slow-twitch	Shorten et al. (2007)
	0.0058 mFmm^−2^	Fast-twitch fiber membrane capacitance	
*σ_i_*	0.893 mSmm^−1^	Internal fiber conductivity	Bryant (1969)
*σ_e_*	0.67 mSmm^−1^	External fiber conductivity	Rush et al. (1963) Schwann and Kay (1957)
*I_s_*	8000 μA/mm^2^	External stimulus current	Assumed

As simulation parameters for solving the Shorten et al. (2007) model using LSODA, a time increment of *Δt *= 0.01 and an absolute error *ε*_tol_ = 0.1^−4^ have been identified to provide a good balance between accuracy and computational speed. The spatial discretization of the muscle fibers is determined by a grid convergence analysis with respect to the conduction velocity. For this purpose, a 32 mm long muscle fiber has been discretized using different grid spacings and the AP propagation is calculated based on the bidomain equations. The AP conduction velocity is determined by the distance that the maximum positive gradient in membrane potential traveled for a given period of time. The results of the computed conduction velocities for different grid point spacings are presented in Table 2.

**Table 2:** **Computed conduction velocities based on different grid spacing for slow- and fast-twitch muscle fibers**.

Discretization (grid points/mm)	Conduction velocities (m/s)	
	Slow-twitch muscle fiber	Fast-twitch muscle fiber
2	1.529	2.174
4	1.488	2.101
8	1.471	2.049
16	1.466	2.033
32	1.466	2.033
64	1.466	2.033
128	1.466	2.033

Based on the computed conduction velocities, a grid point spacing of 0.0625 mm (16 grid points per mm) is assumed to be adequate for solving the bidomain equations for fast- and slow-twitch muscle fibers.

### 3.3 Physiological recruitment of a TA

Choosing an excitatory drive function *E*(*t*), i.e., the temporal recruitment of single motor units and, hence, the motor-unit-associated fibers, the resulting electro-physiological changes within the fibers, and the multi-scale constitutive law, allows to compute the respective forces exerted by the TA. The TA is assumed to undergo only isometric contractions, i.e., the attachment areas of the TA are fixed by assuming zero-displacement boundary conditions. Further, as mentioned earlier, the TA model consists of 903 fibers (cf. Section 3.1), which are distributed over 30 motor units. The total simulation duration is 500 ms. All the parameters of the recruitment model used within this section are listed in Table 3.

**Table 3:** **Values of the coefficients and variables of the recruitment and rate coding model used within this framework**.

Variable	Value	Description	Sources
$F_{i}^{\text{min}}$	8 Hz	Mean firing rate of motor unit *i*	Averaged from several sources (see Section 2.4)
$F_{i}^{\text{max}}$	40 Hz	Peak firing rate of motor unit *i*	Averaged from several sources (see Section 2.4)
Δ*F*^max^	0	Difference in peak firing rate between the smallest and largest MU	Assumed
*c_ν_*	0.2	Coefficient of variation	Fuglevand et al. (1993)
*η*	N(0,1)	Gaussian-distributed random number	Fuglevand et al. (1993)
*RR*	30	Range of recruitment threshold values	Assumed
*g_e_*	2	gain of the motor units	Assumed

Herein, a step function is assumed as a particular choice of *E*(*t*). Every 100 ms, *E*(*t*) increases by 0.25 [the first 100 ms *E*(*t*) is 0]. The time instances at which all the muscle fibers of a single motor units are activated through a nerve signal, are computed based on *E*(*t*). The arrival of a nerve signal at the neuromuscular junction of a skeletal muscle fiber has been modeled by applying a stimulus at the midpoint of the fibers of the respective motor unit as boundary condition within the bidomain equations. The stimulus has been chosen such that the midpoint of the skeletal muscle fiber initiates a depolarization. As one computes first the cellular variables *A*_1_ and *A*_2_ before solving the governing equations of finite elasticity with the already updated and homogenized cellular variables, the coupling between the electrophysiological and the mechanical model can only be considered as weak. There exists no coupling between the mechanical deformation and the cellular model as of yet.

**Figure 3:**
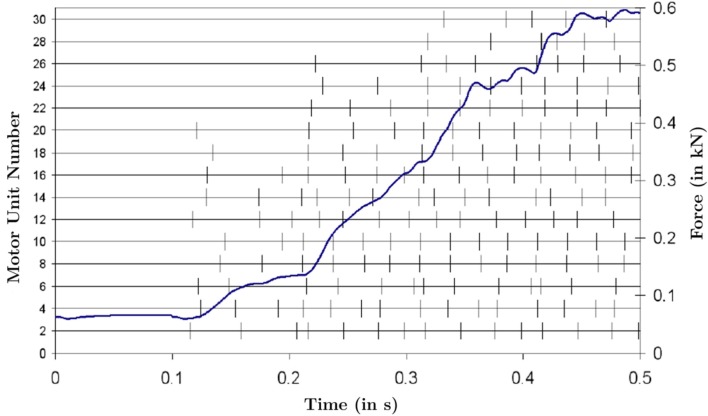
**The stimulation times of every second motor unit are shown with vertical strikes**. It can be seen that the larger motor units become active later in the simulation and the average frequency of all motor units increases throughout the simulation. The sigmoidal shape of the force curve can be seen, with a slow average change in curvature at the beginning and end of the simulation and a relatively linear section in the middle.

The electrophysiological and mechanical problem is solved in 1 ms increments. Based on the finite elasticity solution, one can compute the exerted muscle force at the attachment areas of the TA. Figure 3 depicts the exerted muscle force as a result of the above described excitatory drive function, *E*(*t*). For each (second) motor unit, a separate time line has been included in Figure 3. The small motor unit numbers correspond to the motor units that contain fewer muscle fibers and are sorted in an ascending order (the smallest at the bottom, the largest motor unit at the top). The *y*-axis on the right reflects the exerted muscle force (in kN).

### 3.4 Implications due to different numbers of fibers and motor units

The number of muscle fibers and motor units in a human TA varies greatly, e.g., between 96,000 and 162,000 fibers (Henriksson-Larsén et al., 1983) and 150 ± 43 motor units (McNeil et al., 2005). Modeling each fiber with a spatial resolution of 0.0625 mm would result in more than 50 Mio. computational nodes, at which cellular parameters need to be computed/stored. Moreover, the multi-scale constitutive law will not benefit from such a high resolution after homogenization. Hence, it is of interest to investigate the effects of the number of embedded fibers and the number of motor units on the force-generating capabilities of the TA.

#### 3.4.1 Number of embedded muscle fibers

**Figure 4:**
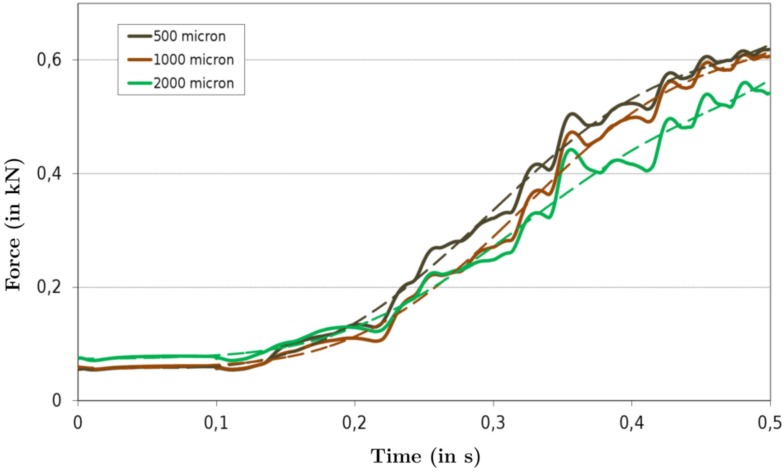
**The force output of TA muscle simulation with mechanical fiber spacings of 2000, 1000, and 500 μm**.

To investigate the effects of changing the number of embedded fibers within the TA geometry, different muscle fiber diameters have been considered, i.e., diameters of 2000, 1000, and 500 μm. Given actual muscle fiber diameters in humans of approximately 80–100 μm (Lexell et al., 1986; Sjöström et al., 1991; Miller et al., 1993), the simulation with the smallest muscle fiber diameter represents already a 1:25 fiber ratio reduction. All simulations in Figure 4 assumed a constant number of motor units, i.e., 30 motor units, while the muscle fiber diameter has been varied. The same motor-unit recruitment protocol as described in Section 3.3 has been applied to compute the resulting muscle forces. The three computed force profiles for muscle fiber diameters of 2000 μm (in green), 1000 μm (in brown), and 500 μm (in black) muscle fiber diameter are plotted in Figure 4. Note, a variation in muscle fiber diameter also requires a recalculation of the muscle fibers association with a particular motor unit. Hence, due to the randomness in selecting muscle fibers to particular motor units, there is the possibility that the location of the muscle fibers belonging to a particular motor unit varies.

Figure 4 contains in addition to the force plots also “smoothed” force profiles (dashed lines of the respective fiber diameter colors). The smoothed force profiles have been computed through a least-squares fit to a 6th-order polynomial. The purpose of fitting the force profile to a smooth polynomial is to investigate the fluctuations of the muscle forces. Hence, the deviation of the force output from the best-fit curve is then given by

(20) $$R^{2}=1-\frac{\sum {\left(y_{k}-{ŷ}_{k}\right)}^{2}}{\sum \overset{2}{\underset{k}{y}}+1n{\left(\sum y_{k}\right)}^{2}},$$

where *y_k_* is the computed muscle force at time *kΔt* with *k* = 1,…, *n* and *n*·*Δt *= 0.5 s and ${ŷ}_{k}$ is the respective value obtained from evaluating the polynomial. For mechanical fiber spacings 2000, 1000, and 500 μm, the *R*^2^ values are 0.9884, 0.9950, and 0.9958, respectively. In Figure 4, the increase in *R*^2^ can be noticed by observing the fact that the force profiles exhibited less variations as the number of fibers increased. The increase in the force as the muscle fiber diameter increases is due to the proportionally larger increase in the number of fibers in the distal section of the muscle with a smaller pennation angle.

#### 3.4.2 Number of motor units

**Figure 5:**
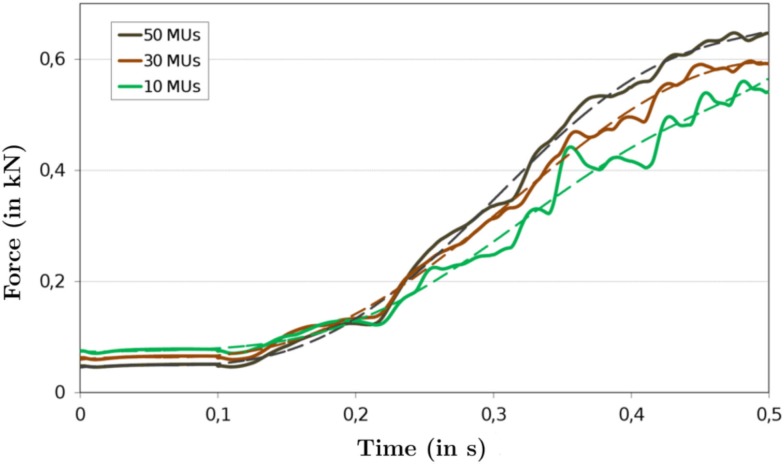
**The force output profiles of the TA with 10, 30, and 50 motor units**. The sigmoidal shapes of the force profile can be clearly seen in the best-fit curves of the plots of 30 motor units and 50 motor units (dashed lines). The change in curvature of the 10 motor-unit simulation is more subtle. The variations in the base line force of the simulations are due to the changing location of the slow and fast fiber types. Increasing the number of motor units increases the maximum value of the force, which is also a result of fiber type location.

Representing the TA with fewer than the actual number of motor units has similar benefits and drawbacks as reducing the number of embedded fibers. Considering fewer motor units reduces the computational cost for calculating the electro-physiological behavior of muscle fibers associated with a motor unit, but increases the possibility of less smooth force outputs. Like above, different simulations with 10, 30, and 50 motor units were carried out. The number of fibers were set to be constant at 903 fibers, i.e., a fiber diameter of 2000 μm (cf. Section 3.3). The excitatory input to the motor-unit pool is the same in each case. The respective force output based on simulations with 10 (in green), 30 (in brown), and 50 motor units (in black) are depicted in Figure 5.

To investigate the smoothness of the different force profiles, the least-square fits are repeated for each muscle force profile and the measure of smoothness, *R*^2^ as defined in equation (20), is computed for each force profile. For the 10, 30, and 50 motor units per muscle, the *R*^2^ values are 0.9884, 0.9967, and 0.9972, respectively. The 30 and 50 motor-unit muscle exhibits an even higher smoothness coefficient than the TA with a 500 μm fiber diameter.

## 4 Discussion

The proposed skeletal muscle modeling framework is capable of replicating many structural and functional aspects in skeletal muscles. The model’s complexity and versatility to examine many different aspects of a skeletal muscle’s function and structure through simulation is certainly the model’s key strength. At the same time, this is also the model’s weakness if muscle-specific simulations are desired. The complexity and versatility requires many input parameters about a skeletal muscle anatomy and functional distribution. The current body of knowledge for some of the input data is quite limited. This is in particular true for data concerning the motor-unit territories and a skeletal muscle’s active and passive mechanical behavior, and, hence, the proper definition of the (multi-scale) constitutive within the theory of finite elasticity. Nevertheless, the forces predicted by the model (i.e., peak forces of 0.5–0.6 kN) agree well with experimental findings, e.g., Brand et al. (1986), Maganaris (2001), or Maganaris et al. (2001).

### 4.1 Uncertainties in motor-unit territory data

The limitations regarding the motor-unit territories stem from the fact that a rigorous description of all the parameters, which define the muscle fiber distribution across the motor units, is not available from the literature (Monti et al., 2001). Due to physical limitations in experiments, it is difficult to spatially analyze more than one motor unit at a time. Further, such analysis are generally based on 2D sections. These two factors combined lead, from an experimentally point of view, to limited insights into motor-unit distributions. Nevertheless, investigation such as those carried out by Bodine-Fowler et al. (1990), Roy et al. (1995), and Monti et al. (2001) can provide enough data for the generic assumptions made in this study.

Although three-dimensional descriptions of motor-unit fiber distributions within a muscle have been attempted, e.g., by analyzing multiple 10–20 μm thick cross-sectional segments of the muscle, the data is essentially only valid for the analyzed muscle. As far as the locations of a motor-unit’s muscle fibers is concerned, there exists a substantial inter-subject variability of the location.

The definition of motor-unit territory weighting centers and the proposed random muscle-fiber-selection algorithm for populating motor units (cf. Section 2.1.2) provides in combination with the proposed continuum-mechanical framework (cf. Section 2.3) an alternative but biophysically based way to analyze the influence of different muscle fiber and motor-unit distributions on its mechanical behavior.

For example, from Figure 5, it becomes apparent that the force output becomes smoother as more motor units are added. In case of adding more motor units, however, there is a greater likelihood that distally located fibers are being activated first. Activating those fibers first would likely cause an increase in force as early activation allows the fibers to obtain a higher total force by the end of the simulation and their distal location means that their pennation angle is less and thus their effect on total force is greater. This is only one example that provides evidence that internal structures of the muscle can significantly contribute to the overall mechanical behavior of the muscle and that more information concerning the force transduction pathways within the muscle should be incorporated.

Further, one can carry out in analogy to the previous conclusion many other simulations aiming to investigate the effect of different muscle fiber and motor-unit distributions on the exerted muscle force. A systematic approach to setup such numerical experiments can lead, together with additional knowledge about the muscle’s function and the purpose of other surrounding tissues, to a reverse-engineering approach of deducing certain (additional and unknown) information on motor-unit distributions.

### 4.2 Limitations in constitutive modeling

Despite the fact that the proposed multi-scale constitutive law does not incorporate many (micro-structural) detailed information on force transduction pathways, one obtains from the overall continuum-mechanical framework muscle forces that are similar to other numerical and physiological studies. Brand et al. (1986), for example, report 535 N as maximal exerted muscle force of the TA and Fukunaga et al. (1997) report for the force-length relationship of TA muscle fibers a maximum force of slightly more than 400 N.

Further, a mathematical validation of the multi-scale constitutive law has been carried out in Röhrle et al. (2008). In this work, Röhrle et al. (2008) showed that the multi-scale constitutive law exhibits for different activation principles, different finite element basis functions, and sequentially refined meshes similar finite element convergence rates as for the mechanical-only problem. This, however, only provides evidence that the homogenization of the cellular parameters for the multi-scale constitutive law have been defined in a mathematical consistent way. A full experimental validation of the coupling method is in fact currently almost impossible as the data required to generate a fully accurate constitutive law does not exist. A large amount of experimental and modeling work needs to be undertaken to fill the gap in the literature regarding the three-dimensional mechanical properties of skeletal muscle tissue in both passive and active states. This work will almost definitely have to begin by looking at the micro-structural linkages between muscle fibers and the role that the epimysium, perimysium, and endomysium plays in modifying both the force output of muscle fibers, and the path of the generated force through the muscle. A more detailed mechanical description of the structure of skeletal muscle is desirable.

Another drawback of the current implementation of the coupling between the zero-dimensional cellular model (the Shorten et al., 2007 model), the one-dimensional fiber model (bidomain equations), and the three-dimensional continuum-mechanical model is the lack of force or length feedback from the continuum-mechanical model to the Shorten et al. (2007) transmembrane model and the recruitment model. The only data needed for each bidomain simulation was the activation timing (from rate coding). This is a considerable drawback, as much of the afferent input to the central nervous systems stems form feedback mechanisms within the muscle and tendon, e.g., muscle spindles and Golgi tendon organs.

### 4.3 The cellular model and muscle fiber recruitment

Feedback of mechanical data to the cellular level can currently not be directly included in the Shorten et al. (2007) model. Moreover, the Shorten et al. (2007) model is only valid for isometric contractions. Therefore the pre- and post-powerstroke concentrations (*A*_1_ and *A*_2_) have been multiplied by the normalized length relationship for active contractions, i.e., $f_{\text{active}}^{{}^{\text{fibre}}}\left(\lambda \right),$ to account for the probability by how much the actin and myosin overlap. The Shorten et al. (2007) model can easily be extended to reflect non-isometric contractions as well. In such a case, the actin-myosin overlap might be directly considered within the cell model and hence would make an additional multiplication with a force-length relationship, which is typically derived from whole-muscle experiments, redundant. While such an extension is a necessary enhancement for a more general case, it would also provide a direct and strong coupling between the cellular and continuum-mechanical model. Another drawback of the Shorten et al. (2007) model within the proposed framework is the fact that the cell model has been precisely parameterized for mouse TA and not for a human TA. However, no parameters for a human skeletal muscle model are available. The bidomain parameters were taken from experimental data on a mouse’s skeletal muscle. The parameters for LSODA were selected to maximize accuracy and minimize computational cost.

The major advantage of the proposed framework is that the motor-unit recruitment model can be replaced, in a straight forward fashion, by any other motor-unit recruitment model. In particular in the field of computational neurophysiology, there exist state-of-the-art motor neuron recruitment models that are no longer phenomenological, e.g., the motor-unit recruitment model used within this framework, but describe neural recruitment based on complex networks of cellular models (e.g., Cisi and Kohn, 2008). The drawback of all existing computational skeletal muscle mechanical models used in the field of computational neurophysiology is the fact that the calculations of the exerted muscle forces are extremely simplified neglecting any anatomical arrangements or mechanical behavior. This is an even more severe restriction, if researchers draw conclusions from their neurophysiological models based on computed muscle forces without considering an anatomically detailed skeletal muscle mechanics model. Linking both modeling approaches, i.e., the systems biological approaches for neural networks and the proposed skeletal muscle mechanics model, can provide a powerful computational framework to gain new insights in neurological disorders associated with muscle diseases. The proposed model is ideal for such research.

### 4.4 Force-velocity relationship

The current framework considers only the special case of isometric contractions of the TA. Throughout this paper, the authors assumed that the force-velocity relationship provides only a very minor contribution within the considered experiments and, hence, can be neglected. Nevertheless, the force-length relationship can be easily implemented within the proposed framework in a number of different ways. Firstly, one can include the force-velocity relationship in a biophysical sense by alternating the Shorten et al. (2007) model. This can be achieved by incorporating biophysical effects altering the crossbridge kinetics as a result of the local contraction velocity. Secondly, the force-velocity relationship can be incorporated into the multi-scale constitutive law in a similar way as the force-length dependency, i.e., by multiplying the active stress tensor, **S**^active^ by a length dependent hyperbolic force-velocity relationship as it has been introduced, for the first time, by Hill (1938) or by Johansson et al. (2000) within a FE framework.

### 4.5 Subject-specific variability

The modeling of the muscle fiber distributions, cellular processes, and its link to a continuum-mechanical skeletal muscle model requires a large set of input variables. To be able to find adequate model parameters, many parameters from different studies had to be combined. In some cases, the parameter did not even originate from humans as ethical measurements of such data in (living) persons would not be able to be conducted. Due to the great inter-subject variability of many of those parameters, it is essentially an impossible task to obtain a realistic set of model parameters for a subject-specific case.

Nevertheless, the proposed skeletal muscle model provides a detailed biophysical skeletal muscle mechanics model. Almost all of the parameters used within the framework are based on some experimental studies. In particular following the described methods for embedding fibers within a three-dimensional geometry and assigning the fibers to a specific motor unit results in anatomically detailed models that could exist in such a way. For instance, the origin and insertion of the skeletal muscle fibers are anatomically realistic and their orientation agrees with published data (Lansdown et al., 2007). The motor-unit distribution is consistent with published physiological data (Monti et al., 2001) and is also similar to the method used by other numerical studies (Yao et al., 2000; Enoka and Fuglevand, 2001). The geometry and the muscle fiber growth algorithm lead to a inherent description of the motor endplate band, which is defined by the center points of all the muscle fibers. The motor endplates within the proposed model form a parabola as it is described in Aquilonius et al. (1984). Hence, the presented framework presents a great resource for exploring and investigating many open questions in skeletal muscle physiology and mechanics in a general and qualitative way.

### 4.6 Validation and human specific parameters

Any physiologically based mathematical model relies on experimentally determined parameters. The comparative scarcity of human data generally means that models of human systems rely on the assumption that meaningful results can be gained when using parameters determined from different species. The benefit of building models of human tissue is that the assumed accuracy of parameters can be tested in a computational setting. Discrepancies between experimentally determined parameters in one species and the computationally predicted results of another species using these parameters can provide an insight into areas where more specific inquiry is needed.

Validation of the framework is therefore of key importance to any model. This work aims to provide a qualitative validation of the model. The trends predicted by the model, e.g., effects of motor-unit location and recruitment effects, fit within known experimental bounds. A more in-depth validation procedure would see a number of subject-specific models created using as many of the subject-specific material properties as possible. In contrast to the simulated approach, a rigorous experimental validation procedure can only be achieved for very simple systems, such as artificially activated *ex vivo* rodent muscle between force transducers. In addition to artificial activation of a muscle, the models predictive capabilities could be tested by accounting for the roles of synergist and antagonist muscle groups similar to the experimental approaches of Maganaris (2001) or Maganaris et al. (2001). Each of the above mentioned validation procedure would require a separate and thorough study. Other validation options include numerical validation procedures to investigate the effects of discretization errors, approximation errors, and errors introduced through the homogenization process of coupling the cellular level with the organ level. A *in silico* validation of the multi-scale constitutive law has been provided previously (Röhrle et al., 2008).

## Conclusion

A novel approach to the three-dimensional modeling of skeletal muscle function is presented. This method has the potential to represent more of the known anatomical and physiological information than other modeling techniques currently available. This approach incorporates cellular physiology, anatomical structure, and functional grouping into a finite elasticity simulation of skeletal muscle. This multi-scale approach allows analysis of the effects of alterations to a large range of physiological and structural parameters, which is important when investigating physiological diseases, mechanical injury, or changes resulting from training and aging. While this framework still requires rigorous validation, it provides one of the most integrated electromechanical model of a skeletal muscle, which is currently available.

## Conflict of Interest Statement

The authors declare that the research was conducted in the absence of any commercial or financial relationships that could be construed as a potential conflict of interest.

## Footnotes

^1^http://models.cellml.org/exposure/159ba2f081022ca651284404f39eeb40/view
